# The Association Between Time-Varying Wall Shear Stress and the Development of Plaque Ulcerations in Carotid Arteries From the Plaque at Risk Study

**DOI:** 10.3389/fcvm.2021.732646

**Published:** 2021-11-18

**Authors:** Kristine Dilba, Dianne H. K. van Dam-Nolen, Suze-Anne Korteland, Anja G. van der Kolk, Mohamed Kassem, Daniel Bos, Peter J. Koudstaal, Paul J. Nederkoorn, Jeroen Hendrikse, M. Eline Kooi, Frank J. H. Gijsen, Anton F. W. van der Steen, Aad van der Lugt, Jolanda J. Wentzel

**Affiliations:** ^1^Department of Cardiology, Erasmus MC, University Medical Center Rotterdam, Rotterdam, Netherlands; ^2^Department of Radiology and Nuclear Medicine, Erasmus MC, University Medical Center Rotterdam, Rotterdam, Netherlands; ^3^Department of Radiology, University Medical Center Utrecht, Utrecht, Netherlands; ^4^Department of Radiology and Nuclear Medicine, CARIM School for Cardiovascular Diseases, Maastricht University Medical Center^+^, Maastricht, Netherlands; ^5^Department of Epidemiology, Erasmus MC, University Medical Center Rotterdam, Rotterdam, Netherlands; ^6^Department of Neurology, Erasmus MC, University Medical Center Rotterdam, Rotterdam, Netherlands; ^7^Department of Neurology, University Medical Centers Amsterdam, Amsterdam, Netherlands

**Keywords:** shear stress (fluid), carotid, ulceration, risk, atherosclerotic cardiovascular disease, MRI, computational fluid dynamics

## Abstract

**Background and Purpose:** Shear stress (WSS) is involved in the pathophysiology of atherosclerotic disease and might affect plaque ulceration. In this case-control study, we compared carotid plaques that developed a *new* ulcer during follow-up and plaques that remained silent for their exposure to time-dependent oscillatory shear stress parameters at baseline.

**Materials and Methods:** Eighteen patients who underwent CTA and MRI of their carotid arteries at baseline and 2 years follow-up were included. These 18 patients consisted of six patients who demonstrated a new ulcer and 12 control patients selected from a larger cohort with similar MRI-based plaque characteristics as the ulcer group. (Oscillatory) WSS parameters [time average WSS, oscillatory shear index (OSI), and relative residence time (RRT)] were calculated using computational fluid dynamics applying the MRI-based geometry of the carotid arteries and compared among plaques (wall thickness>2 mm) with and without ulceration (Mann–Whitney *U* test) and ulcer-site vs. non-ulcer-site within the plaque (Wilcoxon signed rank test). More detailed analysis on ulcer cases was performed and the predictive value of oscillatory WSS parameters was calculated using linear and logistic mixed-effect regression models.

**Results:** The ulcer group demonstrated no difference in maximum WSS [9.9 (6.6–18.5) vs. 13.6 (9.7–17.7) Pa, *p* = 0.349], a lower maximum OSI [0.04 (0.01–0.10) vs. 0.12 (0.06–0.20) *p* = 0.019] and lower maximum RRT [1.25 (0.78–2.03) Pa^−1^ vs. 2.93 (2.03–5.28) Pa^−1^, *p* = 0.011] compared to controls. The location of the ulcer (ulcer-site) within the plaque was not always at the maximal WSS, but demonstrated higher average WSS, lower average RRT and OSI at the ulcer-site compared to the non-ulcer-sites. High WSS (WSS>4.3 Pa) and low RRT (RRT < 0.25 Pa) were associated with ulceration with an odds ratio of 3.6 [CI 2.1–6.3] and 2.6 [CI 1.54–4.44] respectively, which remained significant after adjustment for wall thickness.

**Conclusion:** In this explorative study, ulcers were not exclusively located at plaque regions exposed to the highest WSS, OSI, or RRT, but high WSS and low RRT regions had a significantly higher odds to present ulceration within the plaque even after adjustment for wall thickness.

## Introduction

Carotid atherosclerotic plaque rupture with thrombus formation and artery-to-artery embolism remains one of the leading causes of ischemic stroke (1, 2). Currently, the decision to perform a carotid endarterectomy is determined by the clinical symptomatology and the presence of severe stenosis in the carotid artery (3). However, there is robust evidence that a substantial number of clinical events occur in patients who have a low degree of stenosis, showing that other characteristics of the plaque beyond stenosis may also play an important role (4, 5). Therefore, researchers started to investigate other markers of rupture-prone plaques beyond the degree of luminal stenosis, mainly focusing on plaque composition to identify vulnerable plaques (6). Vulnerable plaques are characterized by a large lipid rich necrotic core (LRNC) covered by a thin fibrous cap and the presence of intraplaque hemorrhage (IPH) (7–10). However, not all vulnerable plaques do rupture and cause symptoms (9).

Wall shear stress (WSS) is the frictional force that flowing blood exerts on endothelial cells and plays a substantial role in plaque initiation and growth (11, 12). Initially, atherosclerotic plaques develop at inner curvature regions or close to side branches, where WSS is low and oscillating (11, 13). WSS oscillations have been defined using the oscillatory shear index (OSI) and relative residence time (RRT) (14, 15). High OSI and RRT were associated with plaque growth in carotid bifurcations (13). Since ulcers were most frequently observed at the upstream part of the stenotic carotid plaques, where WSS is supposedly high, it was hypothesized that high WSS influences plaque destabilization and thus plaque ulceration (16, 17). Furthermore, in coronary arteries it was observed that plaque rupture was also associated with OSI and RRT (18).

Longitudinal studies that investigated the association between WSS and plaque rupture in carotid arteries have hardly been performed, since ulcerations are not frequently captured during the follow-up period. Up till now, only three case studies have been performed reporting controversial results (17, 19, 20). However, they did not evaluate the role of WSS oscillations in relation to plaque ulceration. Therefore, we investigated in a case-control study design the relation between WSS, OSI, and RRT at baseline and new ulceration formation during 2 years of follow-up in patients with mild-to-moderate carotid artery stenosis. Furthermore, a detailed analysis on the ulcer location within the plaque was performed.

## Methods

### Study Population

This study is embedded within the Plaque At Risk (PARISK) study (clinicaltrials.gov NCT01208025) (21). Non-invasive plaque imaging [Ultrasound (US), MDCTA, MRI] was scheduled at baseline in all 240 included patients and in a predefined subset of patients at 2 years follow-up. For this study, we only selected patients that, in addition to baseline MDCTA and MRI, underwent MDCTA at 2 years follow-up.

Cases were defined as patients who developed new plaque ulceration during follow-up. In a previous study (22), we demonstrated that plaques presenting with newly formed ulceration during follow-up have a larger wall volume, percentage LRNC and IPH volume than the cases without ulceration. Moreover, since WSS is dependent on the luminal dimensions of the carotid arteries, the carotid arteries of the selected control patients were chosen to have similar lumen dimensions. The selection procedure was as follows: first, patients with carotid plaques presenting with LRNC and IPH were selected (*n* = 21). Those patients were ranked on minimal lumen diameter and by checking the percentage of IPH, LRNC, and wall volume two control cases were selected per ulcer case. Using this approach, WSS parameters could be compared between patients with (cases) and without new plaque ulceration, independent of known confounding factors. The study was approved by the institutional Medical Ethical Committees. Written informed consent was obtained from each participant before enrollment.

### MDCTA Data Acquisition and Analysis

We performed MDCTA image acquisition by using a standardized protocol, as previously described in the study design article (21). All MDCTA images were transferred to a workstation equipped with dedicated 3D analysis software (Syngo. *via*; Siemens, Erlangen, Germany). This multiplanar reformatting application allowed analysis of carotid arteries in oblique, coronal, and sagittal planes.

The symptomatic carotid artery was analyzed. The degree of luminal stenosis in the carotid artery was measured according to the NASCET and ECST criteria (3, 23). Also, the minimal luminal diameter was established. Plaque surface morphology was evaluated and classified as either ulcerated or non-ulcerated. Plaque ulceration was defined as an extension of contrast material of >1 mm into the atherosclerotic plaque, being visible on at least two perpendicular planes (24).

Plaque surface morphology was evaluated by two trained readers at baseline (B.H. and A.C.v.D) and at follow-up (K.D. and D.v.D.N.). The trained readers (B.H., A.C.v.D., K.D., and D.v.D.N.) were physicians who were first trained on a training set to identify the presence of ulceration on MDCTA. They had to successfully complete this training set before they assessed the ulcerations on the PARISK data set. The third observer who was consulted for consensus in case of no-agreement is a neuroradiologist with > 25 years of experience (A.v.d.L.). Temporal changes in plaque surface morphology were subsequently evaluated by two trained readers (K.D. and D.v.D.N.) by visual comparison of baseline and follow-up images in which a newly formed ulceration was detected.

### MRI Data Acquisition and Analysis

All examinations were performed on 3.0 T whole body MRI scanners [Achieva, Philips Healthcare, Best, The Netherlands; or Discovery MR 750; General Electric (GE) Healthcare, Milwaukee, Wisconsin]. Imaging protocols included five sequences that were comparable between centers. A more detailed description of these sequences has previously been described in the study design article (21). Six observers evaluated the MR images of the symptomatic carotid artery with dedicated vessel wall analysis software (VesselMass; Department Radiology, Leiden University Medical Center, The Netherlands). Observers had to demonstrate good interobserver agreement for all parameters (intraclass correlation coefficient/ kappa values ≥ 0.6) on a validation set that was delineated by experts with > 15 years of experience (M.E.K. and A.v.d.L.), before they could start with delineating the MR images. The observers were blinded to clinical data and other imaging examinations. Firstly, the different MRI sequences were registered, which allowed us to use multisequence imaging criteria to subsequently draw contours of vessel lumen, outer vessel wall and plaque components such as IPH, LRNC, and calcifications (25). The lumen and wall volume were calculated and since, for all patients, the same nine slices (five in the internal and four in the common carotid artery) were used, bias because of different scan ranges that may include different lengths of common carotid artery and internal carotid artery was prevented. Percentage wall volume was calculated as wall volume/(wall volume + lumen volume) * 100%. The relative volume of plaque components to wall volume (outer vessel volume−lumen volume) was also calculated (e.g., percentage IPH = IPH volume/wall volume * 100%).

### Assessment of (Oscillatory) Wall Shear Stress Parameters

**Figure 1:**
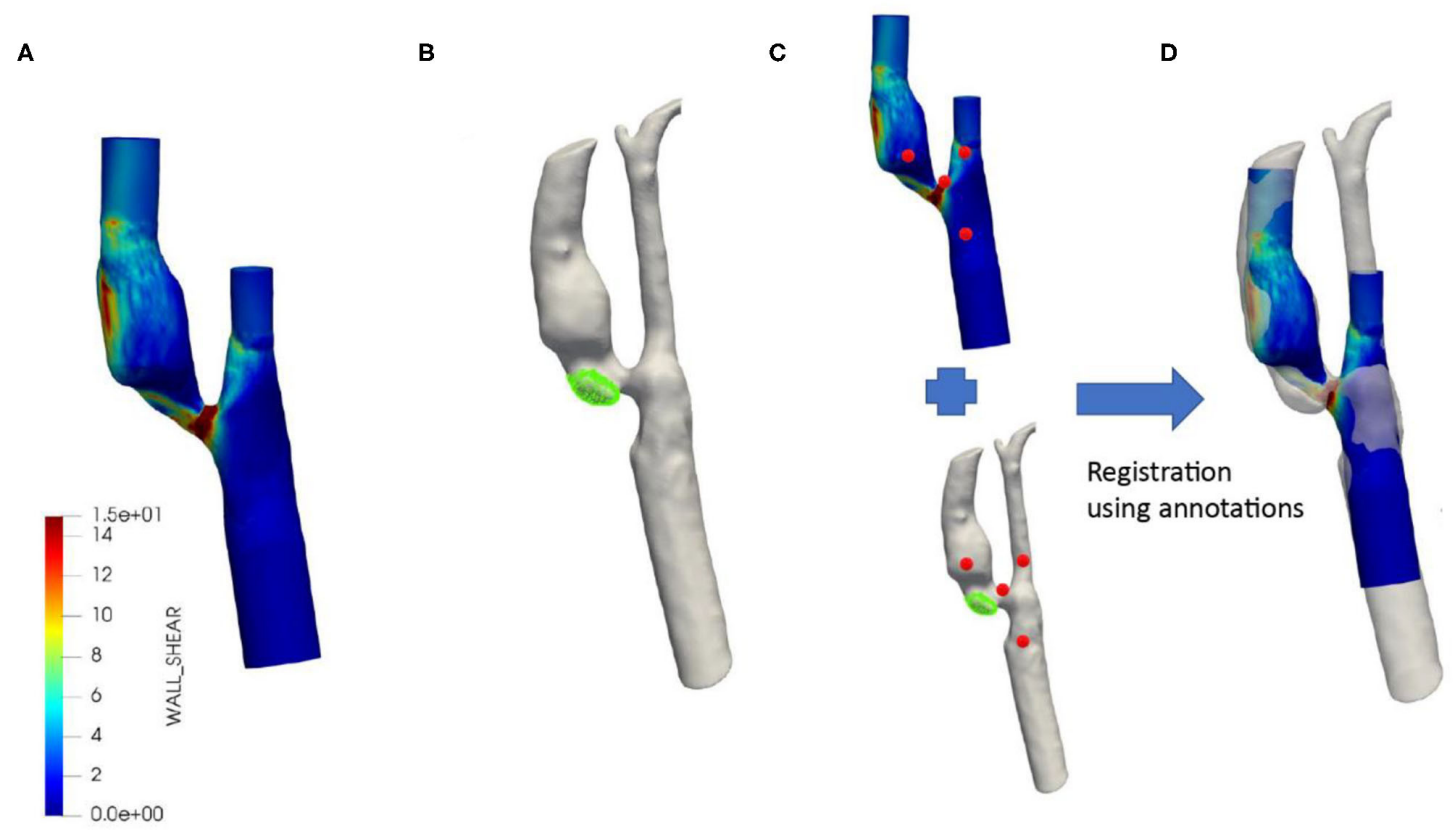
Typical example of co-registered baseline wall shear stress distribution with follow-up MDCTA. WSS distribution over the symptomatic carotid artery based on MRI baseline contours and flow as measured by Duplex **(A)**. Segmented MDCTA follow-up lumen with the new ulcer shown in green **(B)**. Registration between MRI baseline and MDCTA follow-up using annotations (red) **(C)**. Final registration of baseline MRI including wall shear stress and MDCTA with new ulcer **(D)**.

The commercial software MATLAB (The MathWorks, Massachusetts, USA) was used to generate the 3D geometry of the carotid bifurcation using MRI contours obtained from the VesselMass software. The Vascular Modeling ToolKit (VMTK, www.vmtk.org) was used to add flow extensions to the in and outflow boundaries and to smoothen the surface of the bifurcation. The reconstructed geometry was loaded into ANSYS ICEM (version 17.1) to generate a tetrahedral volume mesh with five layers of prism elements at the vessel wall. This mesh was then imported into ANSYS Fluent (version 17.1) to calculate WSS by solving the Navier Stokes equations. The blood was modeled as an incompressible non-Newtonian fluid (Carreau model) with a density of 1,060 kg/m^3^. The arterial wall was assumed to be rigid and a no-slip boundary condition at the wall was applied. At the inlet of the common carotid artery (CCA), a transient flow curve was applied based on the average flow curve (26). For each patient, the flow curve was scaled such that the average flow agreed with the measured flow in the CCA using color Doppler. The flow was obtained by combining the velocity data measured by color doppler with local measurements of the diameter of the artery. This approach was used to be less sensitive to measurement artifacts in the individual patient. A heartrate of 68 bpm was assumed. In addition, using these flows, a Womersley profile was prescribed over the surface of the CCA inlet (27). Outflow ratios for the internal carotid artery and external carotid artery were assumed to be 64 vs. 36%, since the vessels were not severely stenotic (28). The simulation was carried out over two full heart cycles. The first heart cycle was used for initialization, while the second heart cycle was used to compute the (oscillatory) WSS parameters. Each heart cycle was divided into 200-time steps (~4.4 ms dependent on the heart rate). Post-processing of the computational fluid dynamics (CFD) simulation results was done in MATLAB (The MathWorks, Massachusetts, USA). Over the whole vessel, the time averaged WSS (WSS), oscillatory shear index (OSI), and relative residence time (RRT) were calculated (14, 15, 29, 30). The time-averaged WSS is the WSS averaged over the cardiac cycle. OSI represents a ratio between back-and forward going WSS. RRT represents the relative time that a blood particle resides at a certain location at the vessel wall. Figure 1A shows an example of a 3D WSS-map on the lumen surface of the carotid artery.

### Ulcer Segmentation and Registration

In order to study the ulcer location at follow-up in detail with respect to the local oscillatory WSS parameters at baseline, we reconstructed the MDCTA-based lumen and ulcer at follow-up and co-registered this reconstructed 3D geometry to the baseline MRI-based 3D geometry *via* the following steps (Figure 1). First, based on the local Hounsfield Units the vessel lumen and ulcer (320–500 HU) were segmented on MDCTA follow-up images using the open source software *3D slicer* (4.10 version) (31, 32). Subsequently, a four-point rigid registration approach was applied using the *3D slicer* software to register the baseline (MRI) and follow-up geometry (MDCTA). The 1^st^ fiducial point was placed as a landmark on the carina, the 2^nd^ was on the ECA, the 3^rd^ was on the ICA, and the 4^th^ was on the CCA. Using these fiducial points, baseline MRI and follow-up MDCTA lumen geometries were aligned for maximum overlap of the carotid arteries. Finally, the ulcer surface location was projected on the WSS surface mesh using VMTK to determine the exact location of the ulcer within the plaque. This is called the ulcer-site in the plaque and the remainder of the plaque was considered the non-ulcer-site.

### Statistical Analysis

First, clinical characteristics and plaque parameters in cases and controls were compared. Continuous variables were compared with Mann-Whitney U test and categorical data were evaluated using Fisher Exact test. The lumen surface was subdivided into regions (15° x 0.7 mm length) and the local wall thickness and average WSS, OSI, and RRT were calculated over these regions. The plaque regions were defined as the regions with wall thickness > 2 mm.

The analysis was performed at two levels: at patient level and at region level. The analysis was performed at the patient level to identify some extreme (oscillatory) WSS values and wall thickness information characteristic for plaques that develop an ulcer at follow up: the maximum WSS (maxWSS), minimum WSS (minWSS), maximum OSI (maxOSI), maximum RRT (maxRRT), and maximum wall thickness (maxWT). The maximum value was the 95^th^ percentile of all the values. These values were compared for plaques with an ulcer and plaques without an ulcer (control cases). Using the Mann-Whitney *U* test. The same parameters were also determined at the ulcer-site within the plaque in comparison with the remainder of the plaque (non-ulcer-site). Therefore, the plaque surface (3-D surface) was divided into ulcer-site and non-ulcer-site. The methodology to register the ulcer location at follow-up with the (oscillatory) WSS values at baseline is described in detail above. The differences in minimum and maximum WSS, maximum OSI and maximum RRT, and maximum wall thickness between the ulcer-site and non-ulcer-site were compared with a one sample Wilcoxon signed rank test. For the plaques that developed an ulcer at follow-up, the analysis was also repeated at the region level to identify in even more detail the (oscillatory) WSS characteristics that are associated with the location of ulceration at follow-up. Therefore, the regions within the ulcer-site were identified and compared to the regions outside the ulcer site using linear mixed-effect regression models with the patient as a random factor to consider within patient clustering. To compute an odds ratio of oscillatory WSS parameters for the development of ulcer at follow up, the (oscillatory) WSS parameters were categorized in low, mid, and high based on the frequency distribution and logistics mixed-effect regression models with the patient as random factors were applied. The odds ratios are reported with their 95% confidence interval. A value of *p* < 0.05 was considered as significant (two sided). Continuous variables per patient are presented as median with interquartile range. The values presented based on the regional analysis are estimated means and standard error. All calculations were performed using SPSS version 21 (33).

## Results

### Patient Characteristics

**Figure 2:**
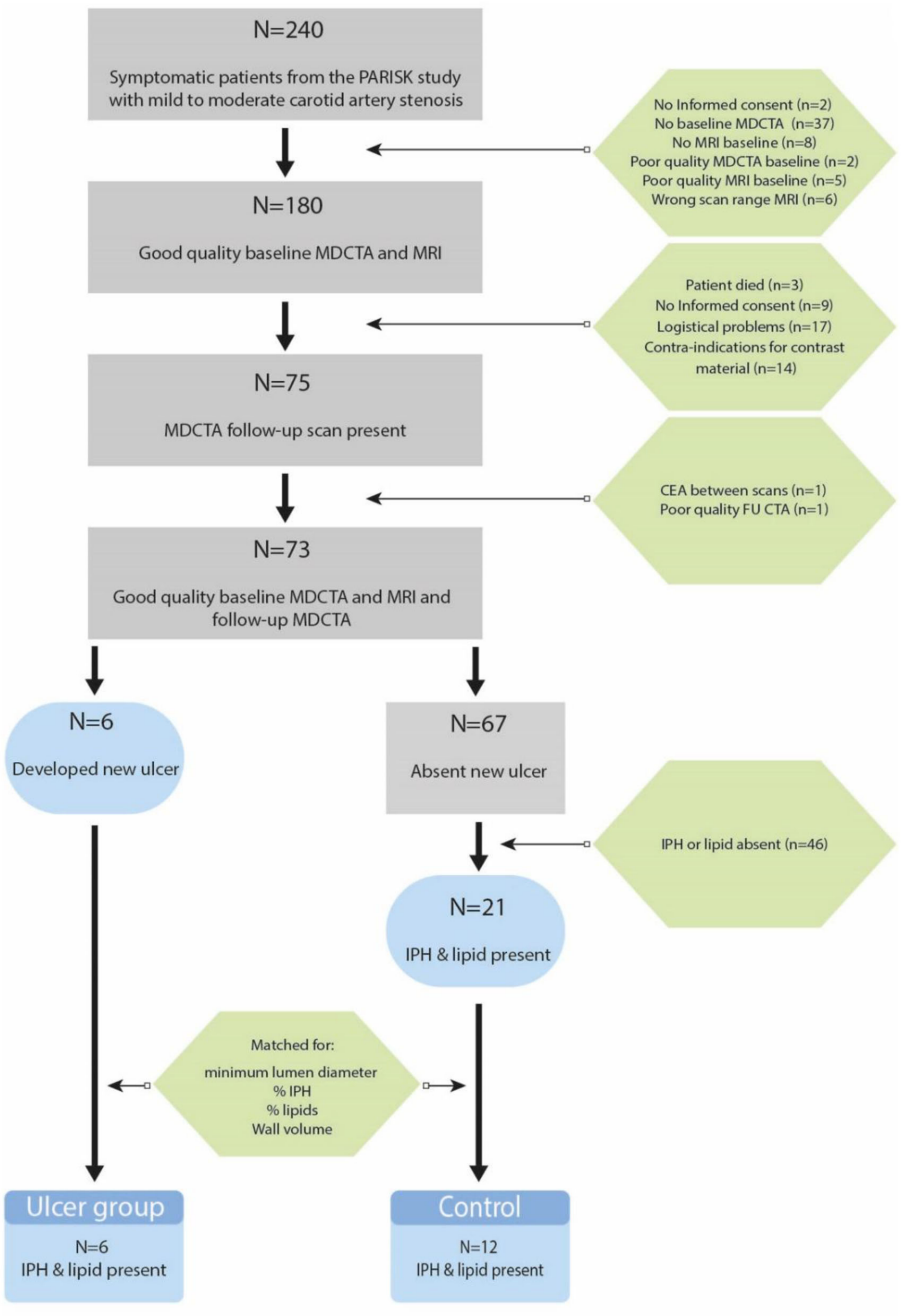
Flow diagram of patients that are included and reasons for exclusion in the final data analysis.

Figure 2 shows a flow diagram of patients included and excluded from the analyses, and the number of patients in the ulcer and control group. Seventy-three patients had good quality CTA and MRI of their carotid arteries to assess atherosclerosis at baseline and 2 years follow-up. New ulcerations developed in six symptomatic atherosclerotic carotid plaques. The control group consisted of 12 patients with matched plaque characteristics. The median age of the study population (*n* = 18) was 70 (62–72) years and 89% of the participants was male. Sixty-one percent of the patients had hypertension, 83% had hypercholesterolemia, and 28% had diabetes mellitus. As anticipated, no differences in minimum lumen diameter, %IPH, %LRNC, and wall volume were observed between the group with and without new ulceration at follow-up (Table 1). However, the matched control cases also did not show differences in other plaque characteristics (Table 1).

**Table 1:** Plaque characteristics in the symptomatic carotid artery with (cases) and without (controls) new ulcerations at follow-up.

	**New ulceration present** **(***n*** = 6)**	**New ulceration absent** **(***n*** = 12)**	***p*** **value**
Total vessel volume (cm^3^)	1.61 [1.53–1.87]	1.55 [1.30–1.86]	0.55
Wall volume (cm^3^)	1.04 [0.97–1.16]	1.04 [0.88–1.15]	0.75
Lumen volume (cm^3^)	0.59 [0.55–0.69]	0.53 [0.45–0.65]	0.25
% wall volume	64 [59–67]	66 [61–68]	0.39
% LRNC volume	23 [16–31]	15 [11–25]	0.15
% calcifications volume	6 [2–9]	7 [5–8]	0.75
% IPH volume	14 [8–24]	11 [7–19]	0.44
NASCET (%)	14 [0–39]	25 [12–35]	0.55
ECST (%)	62 [53–71]	66 [55–69]	0.82
Minimal lumen diameter (mm)	3.4 [2.9–4.8]	3.1 [2.8–4.4]	0.49
LRNC presence	100%	100%	
Calcifications presence	100%	100%	
IPH presence	100%	100%	
Maximal wall thickness (mm)	4.1 [3.6–4.3]	4.0 [3.6–4.5]	0.89
Mean wall thickness (mm)	2.8 [2.6–2.8]	2.7 [2.5–2.9]	0.96

*LRNC, Lipid rich necrotic core; IPH, Intraplaque hemorrhage; NASCET, North American Symptomatic Carotid Endarterectomy Trial, definition of percentage lumen stenosis; ESCT, European symptomatic carotid endarterectomy trial: definition of percentage lumen stenosis*.

### Comparison Between Ulcer Cases and Controls

**Figure 3:**
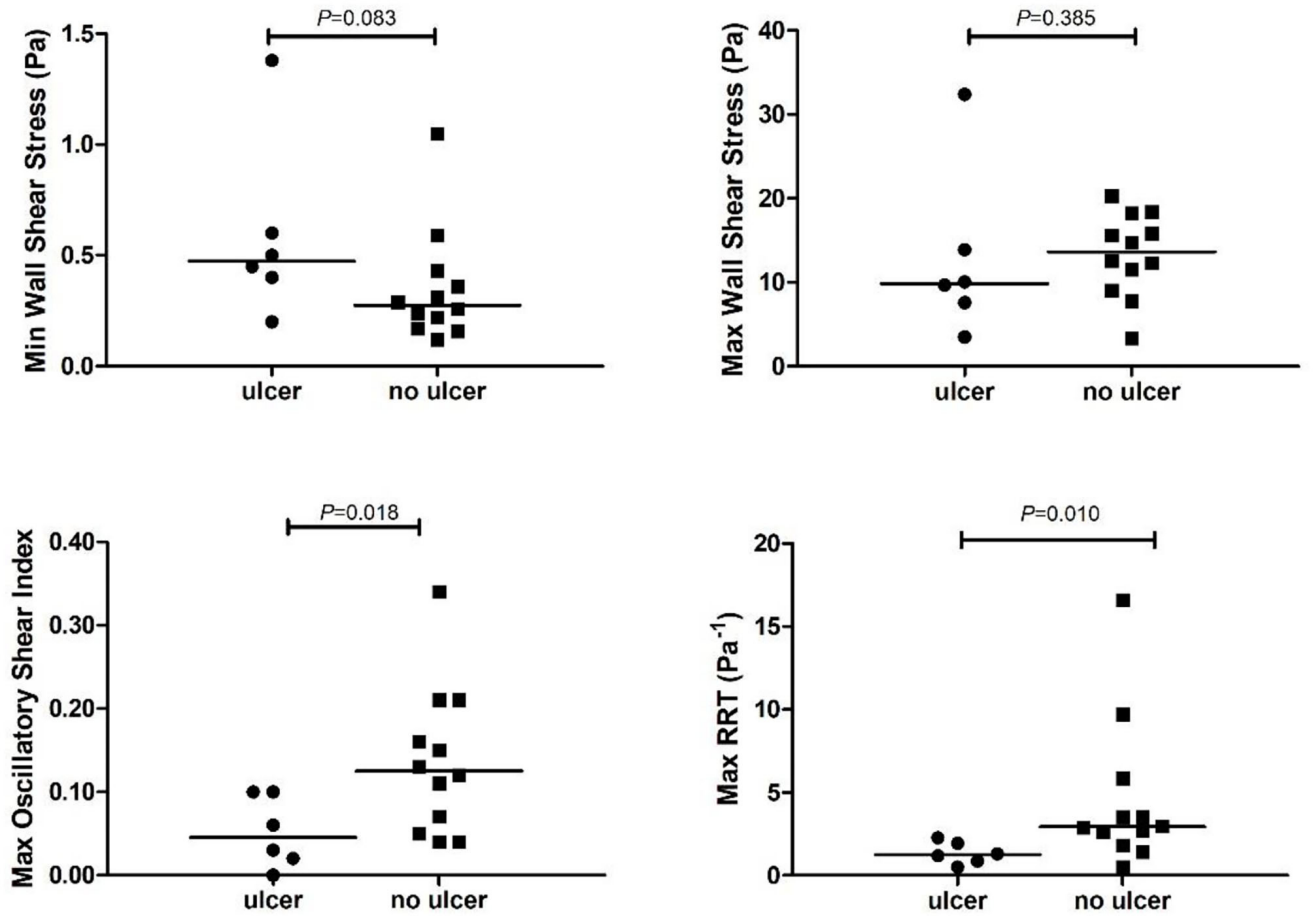
Time varying wall shear stress at the plaque that developed an ulcer (ulcer) vs. the control plaques (no ulcer). RRT, relative residence time. Maximum or minimum time varying wall shear stress values are calculated at the plaque with ulcer or at the plaque without ulcer (no ulcer).

With the small number of plaques that developed a new ulceration after 2 years of follow-up, we could not demonstrate a significant difference in maxWSS compared to plaques that did not develop an ulceration (Table 2, Figure 3). The plaques that developed an ulceration tended to show a higher minWSS at baseline compared to the control group [0.5 Pa (0.4–0.8) vs. 0.3 (0.2–0.4); *p* = 0.083, Figure 3]. Interestingly, the maxOSI was lower for the ulceration group than for the control group [0.04 (0.01–0.10) vs. 0.12 (0.06–0.20); *p* = 0.019, Figure 3]. Regarding RRT, the ulceration group also showed lower maxRRT values compared to control group [1.25 (0.78–2.03) Pa^−1^ vs. 2.93 (2.03–5.28) Pa^−1^; *p* = 0.011, Figure 3, Table 2]. No difference in maxWT was observed [4.1 (3.6–4.25) vs. 4.0 (3.55–4.52) mm, *p* = 0.88].

**Table 2:** Wall shear stress and wall thickness in the symptomatic carotid artery with (cases) and without (controls) new ulcerations at follow-up.

	**New ulceration present** **(***n*** = 6)**	**New ulceration absent** **(***n*** = 12)**	
**Parameters**	**Median [IQR]**	**Median [IQR]**	***p*** **value**
Minimum wall shear stress (Pa)	0.5 [0.4–0.8]	0.3 [0.2–0.4]	0.083
Maximum wall shear stress (Pa)	9.9 [6.6–18.5]	13.6 [9.7–17.7]	0.349
Maximum oscillatory shear index	0.04 [0.01–0.10]	0.12 [0.06–0.20]	0.019
Maximum relative residence time (Pa^−1^)	1.25 [0.78–2.03]	2.93 [2.03–5.28]	0.011
Maximum wall thickness (mm)	4.1 [3.6–4.25]	4.0 [3.55–4.52]	0.888

*Maximum is the 95th percentile of the data except for wall thickness*.

### Comparison Within Ulcerated Plaques: Ulcer-Site vs. Non-ulcer-site

**Figure 4:**
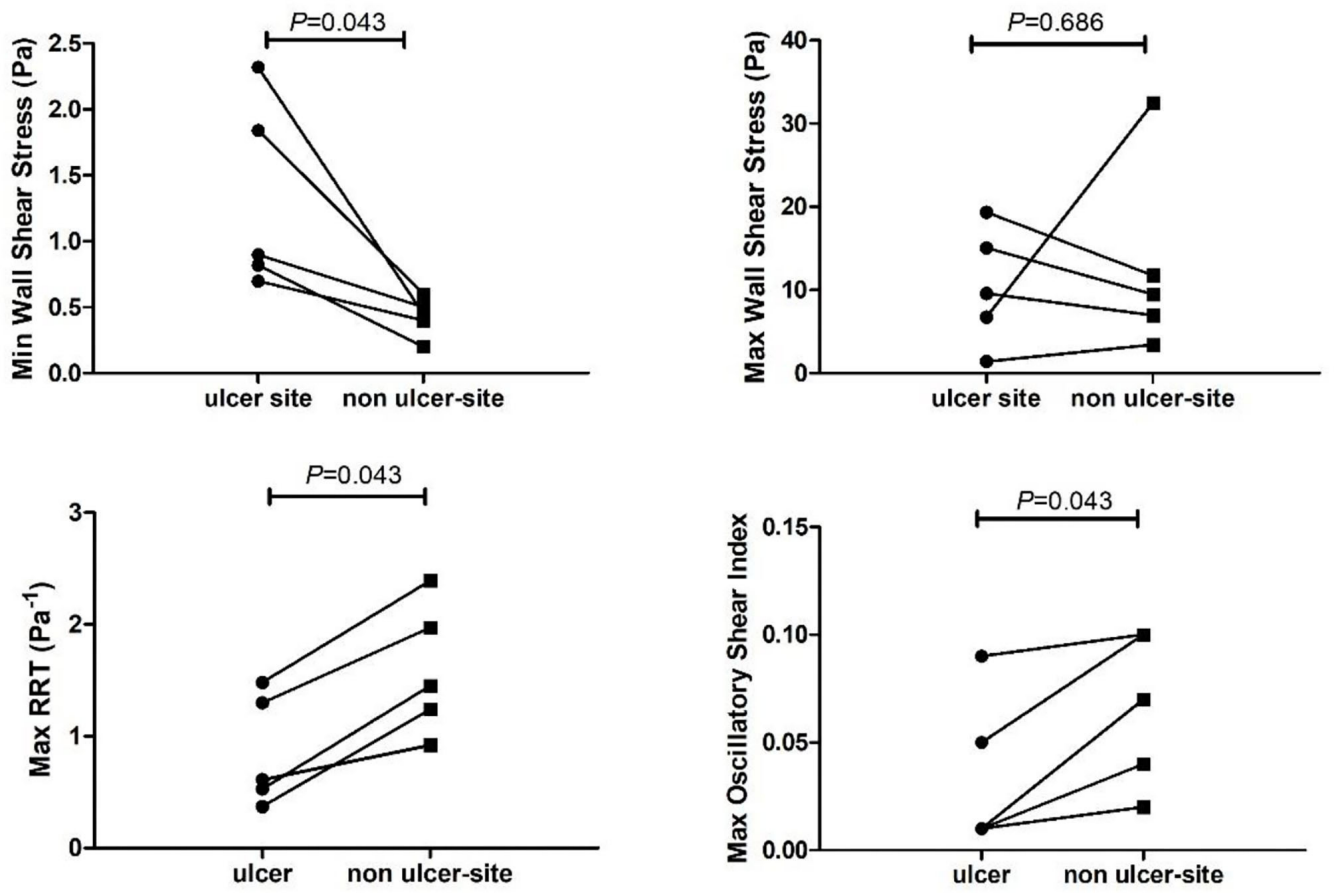
Time varying wall shear stress at the ulcer-site (ulcer) vs. the non-ulcer site (remainder of the plaque) within a carotid artery. RRT, relative residence time.

After registration, we noticed that in one case the ulceration on the follow-up MDCTA was partially located above the scan range on baseline MRI. Therefore, that case was excluded from further analysis. In the remaining five plaques, the minWSS at the ulcer-site was significantly higher than the minWSS at the non-ulcer-site within the plaque (*p* = 0.04) (Table 3, Figure 4). However, for maxWSS, no consistent difference between the ulcer and nonulcer site was observed: in three cases a higher maxWSS and in two cases a lower maxWSS was demonstrated at the ulcer site compared the rest of the plaque (Table 3). The maxOSI and maxRRT were significantly lower at the ulcer site compared to the non-ulcer site (*p* = 0.043 and *p* = 0.043). Furthermore, the maxWT at the ulcer site was not different comparted to the non-ulcer-site [3.8 (3.5–4.1) vs. 4.1 (3.7–4.3) mm, *p* = 0.14; Table 3].

**Table 3:** Comparison of wall shear stress and wall thickness measurements at ulcer site and non-ulcer site.

		**Patient 1**	**Patient 2**	**Patient 3**	**Patient 4**	**Patient 5**	**Patient 6**	**Median**	**P value**
Minimum wall shear stress (Pa)	Ulcer-site	1.8	0.7	0.8	2.3	0.9	NA	0.9 [0.7–2.1]	0.04
	Non-ulcer-site	0.6	0.4	0.2	0.5	0.5	NA	0.5 [0.3–0.6]	
Maximum wall shear stress (Pa)	Ulcer-site	6.7	1.4	15.1	9.6	19.3	NA	9.6 [4.1–17.2]	0.68
	Non-ulcer-site	32.5	3.5	9.5	7.0	11.8	NA	9.5 [5.2–22.1]	
Maximum oscillatory shear index	Ulcer-site	0.05	0.01	0.09	0.01	0.01	NA	0.01 [0.01–0.07]	0.04
	Non-ulcer-site	0.10	0.02	0.10	0.04	0.07	NA	0.07 [0.03–0.10]	
Maximum relative residence time (Pa^−1^)	Ulcer-site	0.61	1.30	1.48	0.37	0.53	NA	0.61 [0.45–1.39]	0.04
	Non-ulcer-site	0.92	1.97	2.39	1.24	1.45	NA	1.44 [1.08–2.18]	
	Non-ulcer-site	2.7	2.0	2.1	2.7	2.4	NA	2.7 [2.5–2.9]	
Maximum wall thickness (mm)	Ulcer-site	3.6	3.8	4.1	3.3	4.1	NA	3.8 [3.5–4.1]	0.14
	Non-ulcer-site	4.1	4.2	4.4	3.3	4.0	NA	4.1 [3.7–4.3]	

*Maximum is 95^th^ percentile, except for the wall thickness*.

### Analysis at Region Level of Ulcer Cases

**Figure 5:**
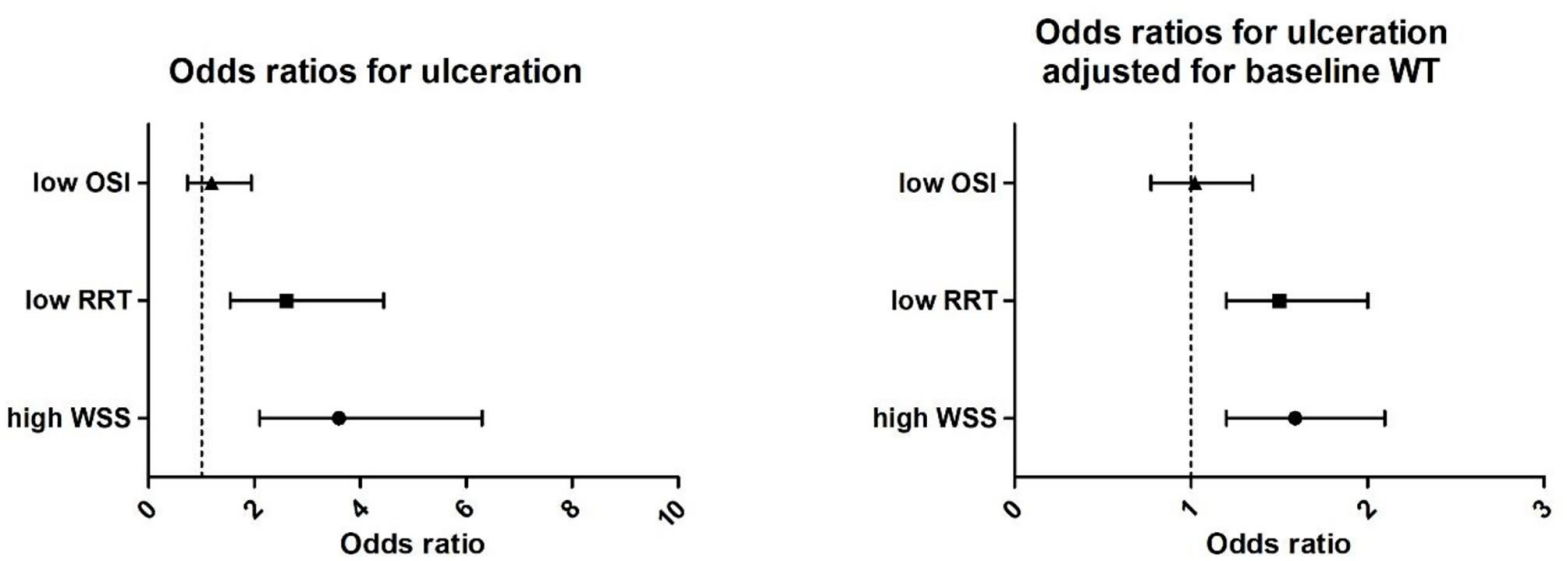
**(Left)** Univariate correlation of high wall shear stress (WSS, > 4.2 Pa), low relative residence time (RRT, < 0.25 Pa^−1^), and oscillatory shear index (OSI < 0.0006) with ulceration presented as odds ratios and **(right)** odd ratios adjusted for baseline wall thickness.

For the ulcer cases, the mean WSS at the ulcer-regions was significantly higher (6.9 ± 40.1 vs. 4.3 ± 40.1 Pa), the RRT was lower (0.37 ± 0.1 vs. 0.65 ± 0.0 Pa^−1^), and WT was higher (3.3 ± 0.4 vs. 2.7 ± 0.4 mm) compared to the non-ulcer regions. For the OSI (0.013 vs. 0.009, *p* = 0.14), no significant differences were found. The odds ratio of low RRT compared to high RRT was 2.6 (CI 1.54–4.44), and high WSS compared to low WSS was 3.6 (CI 2.1–6.3) for the development of ulcers. After adjustment for wall thickness, high WSS and low RRT remained independently associated with ulceration with an odds of 1.59 (1.20–2.1) for high WSS and 1.5 (1.2–2.0) for low RRT. Low OSI was not associated at all with ulceration (Figure 5).

## Discussion

This case-control study investigated the difference in (oscillatory) WSS parameters among plaques that developed an ulcer during a follow-up period of 2 years, and matched control cases. Furthermore, we evaluated these parameters within the plaque comparing the ulcer-site from the non-ulcer site at the patient level and regional level. Our main findings were as follows: plaques that developed an ulcer could not be discriminated from control plaques based on the maximum WSS. However, plaques that developed an ulcer had significantly lower maximum values of OSI and RRT than plaques that did not develop an ulcer. More detailed analysis on (oscillatory) WSS parameters within the plaque demonstrated that at the ulcer location, compared to the non-ulcer-site, the average WSS was higher with higher odds to develop an ulcer at regions exposed to the highest WSS tertile. Moreover, the average RRT was lower, which was also reflected by the lower maximum values in RRT and OSI and higher odds to develop an ulcer at regions exposed to the lowest RRT tertile. Besides, ulcers developed at the thicker portion of the plaque. High WSS and low RRT remained significantly associated with ulceration after adjustment for wall thickness.

Several studies showed that carotid plaques containing IPH are associated with a high risk on future cardiovascular events (10). Furthermore, in an earlier study, we demonstrated that ulceration, a precursor of events, was not only associated with the presence of IPH but also with LRNC (22). Since plaques that contain IPH are likely to be exposed to high WSS (34) and high WSS triggers molecular pathways involved in fibrous cap thinning (35), it was hypothesized that high WSS plays a role in plaque rupture. Therefore, to study the independent influence of WSS on plaque ulceration, we opted for a case control study design in which both plaques that developed an ulcer and control plaques contained IPH along with LRNC with similar lumen dimensions. By applying this study design, for this low number of plaques, we could not demonstrate significant differences in maximum values of WSS between plaques that developed an ulcer and those that did not. However, a more detailed analysis using the individual regions proved that the average WSS on the ulcer-site was higher compared to the remainder of the plaque. Also, the higher WSS tertile (>4.3 Pa) proved to be associated with ulceration. So, this might imply that even though ulcers do not always develop at the location exposed to the highest WSS within the artery at baseline, high WSS might still be instrumental in predicting ulcer location.

Three longitudinal case studies were performed that investigated the role of WSS in plaque ulceration within the ulcerated plaques (17, 19, 20). In the current study, we did not only study one case but had the possibility to investigate the development of five ulcers and their relation to baseline WSS. Interestingly, the three studies reported before described the same variation in WSS exposure at the ulcer site (Table 3). Groen et al. (17) and Wu et al. (19) reported higher WSS at the ulcer site compared to the non-ulcer site, whereas Leach et al. (20) reported lower WSS at the ulcer site. The predictive value of high WSS (>4.3 Pa) for ulceration is in agreement with a study in coronary arteries that also demonstrated that high WSS (>6.56 Pa) is associated with ulceration using univariate analysis (18). However, in that study multivariable analysis demonstrated that wall shear stress gradient is a stronger predictor than time average WSS (18). Other studies in coronary arteries already showed the association between high WSS (>4.71) and coronary events (36, 37).

Next to the observations on high WSS, we noticed that the minimal WSS was 1.7x−5x higher than the minimal WSS over the plaque. This observation implies that ulcers in our study population do not develop at the absolute minimum WSS at the plaque. Low WSS is known for its involvement in plaque progression (11) and lipid accumulation (12) and is therefore thought to potentially play a role in plaque destabilization and rupture. However, in this study low, WSS had lower odds compared to high WSS to develop an ulcer.

Our analysis also showed that plaque ulcerations particularly occur at the thicker part of the plaque. Although wall volume was shown to be a predictor for ulceration (38), no studies investigated the wall thickness as a predictor of the preferred site of plaque ulceration within the plaque. Therefore, we adjusted our analysis for local wall thickness and, accordingly, both wall thickness and high WSS were independently associated with ulceration (Figure 5).

A possible explanation for our findings could be that the thicker part of the plaque is more diseased since it more often contains plaque components such as IPH or LRNC that are known to be associated with plaque rupture (39). If the endothelial cells are exposed to high WSS but not necessarily the highest WSS, this leads to plaque destabilization and, finally, plaque rupture. Therefore, *local* plaque morphology in combination with hemodynamics parameters might be the key in identifying regions at risk for rupture.

Interestingly, while also exploring oscillatory WSS metrics known to be associated with plaque rupture in coronary arteries (18), potentially through regression of fibrous tissue (40), we found that the maximum RRT and OSI values were significantly lower in plaques that developed an ulcer compared to the control plaques. This means that high oscillations in the flow are not a prerequisite for plaque rupture. While studying the odds ratio for ulcer development, only low RRT and not OSI was associated with future ulceration. In a coronary artery study on ulceration, univariate analysis of plaque rupture proved that low OSI had a predictive value for ulceration. However, OSI was not any more significant in multivariate analysis with other oscillatory WSS parameters (18). Therefore, taking these observations together, it seems that OSI is not so strongly associated with ulceration. Future studies are needed to investigate which of the hemodynamic parameters is the strongest predictor.

We compared our data mostly to other work in coronary arteries. However, coronary arteries present with slightly different vulnerable plaque morphology, anatomy, and related hemodynamics (41) which might also explain some of the discrepancies with other studies. Vulnerable carotid plaques as compared to coronary plaques are characterized by a thicker fibrous cap, a higher prevalence of intraplaque hemorrhage, a lower prevalence of plaque erosion, and finally, a higher prevalence of calcified nodules. Furthermore, carotid arteries have a distinct anatomy with a bifurcation of two almost equally sized arteries and a bulb region. In particular, this bulb region is notorious for local oscillatory WSS behavior that is less present in coronary arteries (42).

The strength of this case control study is the longitudinal study design that allowed us to link the WSS at baseline to ulcer formation in the follow-up period. Previous cross-sectional studies showed that ruptured plaques were exposed to high WSS and wall shear stress gradient compared to non-ruptured plaques (18, 43, 44). While comparing our data with those studies, we have to be aware that in contrast to earlier cross-sectional studies in which the investigator tried to reconstruct pre-rupture lumen geometry and determine WSS on the reconstructed lumen, we used the true baseline 3D lumen geometry in our WSS analysis. In fact, in those cross-sectional studies, the 3D-reconstruction of the pre-rupture geometry might not be fully representative for the true baseline geometry since, at the ulcer site, a large part of plaque is washed out and plaques are perhaps smaller. Therefore, since WSS measures and ulcer location are studied in the pre-ulcer 3D geometry, these cross-sectional studies might serve in finding pathophysiological explanations for plaque ulceration rather than parameters to predict plaque ulceration in the future. Taken together, the association between WSS and plaque ulceration using a cross-sectional study design might show different results from the ones obtained with a longitudinal study design. On top of that, we cannot rule out that the local WSS changed in the follow-up period so that it is still possible that the highest WSS in an artery are precursors of future plaque rupture.

This study has several limitations. Since only six ulcers developed in the studied patients, we were consequently restricted to those cases. However, the case-control study design allowed us to correct for multiple known risk factors along with the study of (oscillatory) WSS. Obviously, our findings on the association between WSS, OSI, and RRT should be confirmed in a larger cohort study. Another limitation is the registration of the (oscillatory) WSS based on MRI to the location of ulceration as assessed by MDCTA to study the WSS at the site of the ulceration. We opted for this approach to benefit from the advantages of both image modalities. Ulceration is proven to be the best identified using MDCTA (45, 46) whereas MRI delivers much more detailed information on plaque composition (47). Although we cannot rule out possible influence of misregistration in this detailed WSS analysis, the analysis comparing cases and controls that do not use this registration also resulted in significant differences and in the similar direction of maxRRT and maxOSI. Because of the careful registration, we revealed that one ulcer was partly located outside of the MRI range. Therefore, we included only five cases for the detailed analysis. However, for the whole plaque analysis, comparing time-dependent WSS parameters with control plaques, we did include that sixth case. Since the ulcer region, in that analysis was not included, the plaque analysis might not be fully representative for that plaque. Therefore, the plaque analysis was repeated for 5 cases with the 12 controls. Also, if we analyzed these 5 cases only, the baseline geometric parameters were similar to the 12 controls, and maxRRT and maxOSI remained significant (Supplementary Table I, Supplementary Table II).

## Conclusion

In this study, symptomatic carotid artery plaques that developed a new ulcer during follow-up were investigated. These plaques were also compared to control cases. We demonstrated that plaques that undergo ulceration cannot be distinguished from the control cases based on the maximum WSS values. More detailed analysis on the ulcer location showed that ulcers do not exclusively develop at plaque regions exposed to the highest WSS, OSI, or RRT. However high WSS and low RRT had a significantly higher odds to present ulceration at 2 years follow up within the plaque even after wall thickness adjustment. These data might imply that high WSS and low RRT in combination with local, underlying morphology, and plaque composition predicts future ulcerations. More studies are needed to confirm our findings.

## Data Availability Statement

The datasets presented in this article are not readily available because the data is generated in a consortium. Approval by the consortium is needed before the data can be shared. Requests to access the datasets should be directed to j.wentzel@erasmusmc.nl.

## Ethics Statement

The studies involving human participants were reviewed and approved by Institutional Medical Ethical Committee. The patients/participants provided their written informed consent to participate in this study.

## Author Contributions

JW, KD, and AL contributed to conception and design of the study. MK, DD-N, JH, PK, PN, AL, AS, AK, MEK, and KD organized the database and/or performed general analysis. S-AK performed the computational modeling. JW, FG, KD, and DB interpreted the data. KD and JW performed the statistical analysis and wrote the first draft of the manuscript. All authors contributed to manuscript revision, read, and approved the submitted version.

## Funding

This research was performed within the framework of the Center for Translational Molecular Medicine (www.ctmm.nl), project PARISK (Plaque At RISK; Grant 01C-202) and supported by the Dutch Heart Foundation. KD was in part supported by STW project number 10813.

## Conflict of Interest

The authors declare that the research was conducted in the absence of any commercial or financial relationships that could be construed as a potential conflict of interest.

## Publisher's Note

All claims expressed in this article are solely those of the authors and do not necessarily represent those of their affiliated organizations, or those of the publisher, the editors and the reviewers. Any product that may be evaluated in this article, or claim that may be made by its manufacturer, is not guaranteed or endorsed by the publisher.
