# Supplementary material for: The Association Between Time-Varying Wall Shear Stress and the Development of Plaque Ulcerations in Carotid Arteries From the Plaque at Risk Study
Source: Front Cardiovasc Med. 2021 Nov 18;8:732646. doi: 10.3389/fcvm.2021.732646 (PMC8636734; doi:10.3389/fcvm.2021.732646)
Supplement: Supplementary file 1 [file Data_Sheet_1.PDF]

SUPPLEMENT DATA

Supplement Table I. Plaque characteristics in the symptomatic carotid artery with (cases) and without (controls) new ulcerations at follow-up.

	New ulceration present (n=5)	New ulceration absent (n=12)	p value
Total vessel volume (cm^3)	1.62 [1.53-1.90]	1.55 [1.30-1.86]	0.46
Wall volume (cm^3)	1.05 [1.00-1.18]	1.04 [0.88-1.15]	0.56
Lumen volume (cm^3)	0.58 [0.53-0.73]	0.53 [0.45-0.65]	0.32
% wall volume	65 [60-67]	66 [61-68]	0.53
% LRNC volume	22 [14-27]	15 [11-25]	0.29
% calcifications volume	4 [2-10]	7 [5-8]	0.46
% IPH volume	13 [7-19]	11 [7-19]	0.83
NASCET (%)	27 [0-41]	25 [12-35]	1.00
ECST (%)	67 [50-71]	66 [55-69]	1.00
Minimal lumen diameter (mm)	3.2 [2.7-4.8]	3.1 [2.8-4.4]	0.53
LRNC presence	100%	100%	
Calcifications presence	100%	100%	
IPH presence	100%	100%	
Maximal wall thickness (mm)	4.1 [3.7-4.3]	4 [3.6-4.5]	0.92
Mean wall thickness (mm)	2.8 [2.6-2.9]	2.7 [2.5-2.9]	0.83

LRNC: Lipid rich necrotic core; IPH: Intraplaque hemorrhage; NASCET: North American Symptomatic Carotid Endarterectomy Trial: definition of percentage lumen stenosis; ESCT: European symptomatic carotid endarterectomy trial: definition of percentage lumen stenosis.

Supplement Table II. Wall shear stress and wall thickness in the symptomatic carotid artery with (cases) and without (controls) new ulcerations at follow-up.

	New ulceration present (n=5)	New ulceration absent (n= 12)	
Parameters	Median [IQR]	Median [IQR]	p value
Minimum wall shear stress (Pa)	0.45 [0.30-0.55]	0.27 [0.18-0.41]	0.195
Maximum wall shear stress (Pa)	10.0 [5.54-23.13]	13.6 [9.65-17.65]	0.506
Maximum oscillatory shear index	0.055 [0.0255-0.0995]	0.12 [0.056-0.195]	0.048
Maximum relative residence time (Pa^{-1})	1.31 [1.03-2.11]	2.93 [2.03-5.28]	0.027
Maximum wall thickness (mm)	4.10 [3.7-4.30]	4.00 [3.55-4.52]	1.000

Maximum is the the 95th percentile of the data except for wall thickness
